# Study design of PerfectFit@Night, a workplace health promotion program to improve sleep, fatigue, and recovery of night shift workers in the healthcare sector

**DOI:** 10.1186/s12889-022-13206-9

**Published:** 2022-04-18

**Authors:** Fleur van Elk, Suzan J. W. Robroek, Sonja Smits-de Boer, Tessa A. Kouwenhoven-Pasmooij, Alex Burdorf, Karen M. Oude Hengel

**Affiliations:** 1 Department of Public Health, Erasmus University Medical Center, PO Box 2040, 3000 CA Rotterdam, The Netherlands; 2 Occupational Health Service, Erasmus University Medical Center, Rotterdam, The Netherlands; 3 VitAll, Rotterdam, The Netherlands; 4 Department of Work Health Technology, Netherlands Organisation for Applied Scientific Research TNO, Leiden, The Netherlands

**Keywords:** Workplace, Night shift, Healthcare, Individual, Environment, Sleep, Lifestyle

## Abstract

**Background:**

Healthcare workers need to be at work 24 h a day to ensure continuity of care in hospitals. However, shift work - particularly night shifts - can have negative acute and long-term effects on health and productivity due to disturbances in the circadian rhythm. Shift work is also associated with unhealthy lifestyle behaviors such as poor sleep hygiene and diet. The PerfectFit@Night intervention aims to improve sleep and recovery, and reduce fatigue, and therewith contribute to sustainable employability of healthcare workers. The current study describes the intervention and the evaluation and implementation.

**Methods:**

The study population will consist of healthcare workers, nurses and physicians, with night shifts in a large Dutch academic hospital. The intervention consists of individual and environmental intervention elements: i) an e-learning for healthcare workers to increase knowledge and awareness on a healthy lifestyle during night shifts, ii) a powernap bed to take powernaps during night shifts, iii) the availability of healthy food at the department during night shifts, iv) a workshop on healthy rostering at the level of the department, and v) individual sleep coaching among the high risk group. In a longitudinal prospective study, data will be collected 1 month before the start of the intervention, in the week before the start of the intervention, and three and 6 months after the start of the intervention. The primary outcomes are sleep, fatigue, and need for recovery. The implementation process will be evaluated using the framework of Steckler and Linnan. Cost-benefit analyses from the employers perspective will be conducted to understand the possible financial consequences or benefits of the implementation of PerfectFit@Night.

**Discussion:**

The feasibility and effectiveness of this workplace health promotion program will be investigated by means of an effect, process and economic evaluation. If proven effective, PerfectFit@Night can be implemented on a larger scale within the healthcare sector.

**Trial registration:**

Netherlands Trial Register trial number NL9224. Registered 17 January 2021.

## Background

To ensure continuity of care in hospitals, healthcare workers need to be at work 24 h a day. Consequently, the healthcare sector has one of the highest proportions of night workers. In the Netherlands, one out of five workers in the healthcare sector works night shifts [1]. However, studies show that shift work, particularly night shifts, can have a negative effect on the health and productivity of workers, which is mostly due to disturbances in the circadian rhythm and a lack of sleep [2, 3]. Night work could lead to acute problems such as digestive problems, fatigue, loss of sleep [4], and a higher susceptibility to respiratory infections [5], but also to long-term problems, such as chronic stress and chronic sleep loss [6], and lifestyle-related diseases such as diabetes type 2 [7], cardiovascular diseases [8], mental health problems [9, 10], and all-cause mortality [11].

Shift work is also associated with unhealthy behaviors, such as poor sleep hygiene [12], and changes in timing, quantity, and quality of food intake [13]. Unhealthy behaviors are risk factors for several health problems related to shift work, such as diabetes type 2 [14] and cardiovascular diseases [15, 16]. According to the World Health organization (WHO), preventing or delaying these diseases include targeting lifestyle behavior [15].

Because of the high proportion of healthcare workers with night work, interventions specifically aimed at this group of workers are needed. Especially since it is not possible to avoid night shifts in the healthcare sector. Recent reviews of health promotion interventions among night workers conclude that the effectiveness of these interventions is inconclusive and based on heterogeneous interventions, with some interventions showing modest positive effects [17–21]. The interventions that were assessed in these reviews concern a variety of exposures to bright light, policies and timing schedules regarding napping, types and schedules of physical exercise, and healthy meal offers. Besides the differences between studies assessed in these reviews, a moderate positive health and performance effect was found for napping during night shifts [18, 19].

To contribute to an improvement of health and sustainable employability among night workers in the healthcare sector, the PerfectFit@Night intervention is developed to improve sleep and recovery, and to reduce fatigue. We will evaluate the effectiveness of the intervention on sleep, fatigue, and recovery, as well as the implementation. The current paper describes the PerfectFit@Night intervention and the design of the effect, process, and economic evaluation.

## Methods

The PerfectFit@Night intervention is developed by a needs assessment combining existing literature with a participatory approach to ensure that individuals’ needs and preferences, such as individual characteristics (sleep, dietary behavior, personal factors) and the social and physical context (work-private life, roster, work environment), are also taken into account.

Based on the needs assessment, the following program objectives emerged: i) to increase the knowledge and awareness on working healthy during night shifts, ii) to accept taking powernaps and actually take powernaps during night shifts, iii) to have insight in and availability of healthy food/snacks during night shifts, iv) to have knowledge on healthy rostering, and, additionally, v) to provide sleep coaching for night workers with existing sleep problems and fatigue.

### Intervention elements

**Fig. 1:**
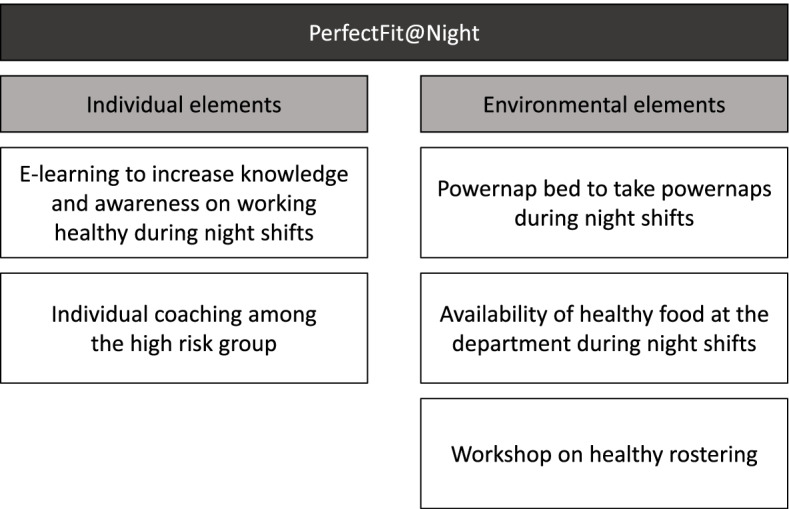
Intervention elements of the PerfectFit@Night intervention divided into individual and environmental elements

Based on the needs assessment and program objectives, the intervention consists of the following individual and environmental elements (see Fig. 1): i) an e-learning for night workers to increase knowledge and awareness on a healthy lifestyle during night shifts, ii) a powernap bed to take powernaps during night shifts, iii) the availability of healthy food/snacks at the department during night shifts, iv) a workshop on healthy rostering at the level of the department (for roster makers), and v) individual sleep coaching among the high risk group.

PerfectFit@Night consists of individual and environmental intervention elements (see Fig. 1). This section describes the intervention elements and the behavioral and environmental components using a behavior change model. For the individual intervention elements, the attitude, social influence, and self-efficacy (ASE) model [22] is used, and for the environmental elements the Analysis Grid for Environments Linked to Obesity (ANGELO) framework [23] is used. The ASE model assumes the need to have the intention to behave a certain way [22]. Attitude, social influence (subjective norms), and self-efficacy influence this intention. The ANGELO framework can be used to describe environmental factors that influence behavior. These factors are divided into physical, economic, political, and sociocultural factors [23].

#### E-learning for night workers

To increase knowledge and awareness on working healthy during night shifts, an interactive e-learning is developed for healthcare workers with night shifts. This e-learning focuses on the effects and possible risks of night work, and it proposes additional information and advice on how to decrease these risks. The e-learning consists of three short modules of approximately ten to fifteen minutes each, of which each on a specific topic, namely the impact of night work on health, sleep and powernaps, and nutrition and physical activity. The e-learning concludes with a test for self-assessment. The night workers receive credits (Continuing Medical Education) to motivate them to start and complete the e-learning.

In line with the ASE model, the e-learning targets i) beliefs about, for example, the working mechanisms of powernaps to change attitude, ii) requirements in the social setting (e.g., subjective norms, social support, role models) to change social influence, and iii) challenging night workers to set goals and experiment to enhance self-efficacy.

#### Powernaps

In order to accept and encourage healthcare workers to take a nap during night shifts, powernap beds were purchased. Following the ANGELO framework, installing a powernap bed at the department, which is placed in a quiet and suitable room (i.e., physical factor), is paid by the participating departments or hospital (i.e., economic factor), and it is allowed by the management to take a powernap (i.e., political and cultural factor). At the individual level, sociocultural factors are role models, social support from colleagues and the management, and social norms regarding powernaps. It is known that attitude towards and acceptance of taking naps during night shifts is challenging. Therefore, the e-learning provides information about the working mechanisms of powernaps and encourages the intention to use powernaps. Besides, examples of good practices in other departments and hospitals are given, and workers of the department are involved as ambassadors (role models).

#### Availability of healthy nutrition at work

To encourage healthy nutrition, healthcare workers with night shifts should have insight in healthy nutrition, and healthy food needs to be offered by the employer. The night workers are informed about the best timing to consume specific nutritional elements through the e-learning module, but also by flyers and a webinar. This information is established in a collaboration with a dietician who specializes in chrono nutrition, based on the latest insights from research and experiences. Following the ANGELO-framework, healthy food is made available during night shifts by providing products (i.e., physical factor) that is paid by the department (i.e., financial factor). The heads of the departments agreed to provide and pay for the food (i.e., political factor). At the individual level, sociocultural factors that are a condition for change in diet to occur are role models (ambassadors), social support from colleagues and the management, and social norms.

#### Workshop on healthy rostering

Having insight in healthy rostering at the level of the department is targeted by a workshop that focuses mainly on staff members, team leaders, and managers dealing with the rosters. The workshop will be tailored to the department. Even though research shows that forward rotating rosters have the best effects, the optimal roster for shift workers remains unknown. The workshop aims to motivate roster makers to have conversations with their workers about the rosters. In line with the ANGELO-framework, the workshop aims to start a conversation at the workplace about changing the rosters (i.e., political factor), and motivate to actually make changes to the rosters (i.e., physical factor). Also important is the acceptance from the management and the workers that the rosters change (i.e., socio-cultural factor), and a sufficient amount of workers to change the rosters (i.e., financial factor). The social context with financial and human resources consequences will also be addressed.

#### Coaching among risk group with existing large sleep problems

The previously described intervention elements are mainly focused on universal prevention of risks due to night work. Additionally, night workers with existing large sleep problems and fatigue are offered sessions with a sleep coach to reduce these problems (indicated prevention). Night workers that score low on subjective global sleep quality (assessed using Pittsburgh Sleep Quality Index (PSQI) [24]) and high on fatigue (assessed using the Short Fatigue Questionnaire (SFQ) [25]) at the baseline measurement are offered coaching sessions, online or face-to-face. The coaches provide evidence-based methods, based on cognitive behavioral therapy insomnia (CGTi), to target the individual problems of the night workers. Participants who accept the invitation for coaching sessions are offered 2–3 sessions of 30–40 min, based on the needs of the individual. During these sessions, the coach proposes the relevant topics to be discussed based on the individual needs and issues of the participant. Before the first session, participants are asked to fill out a screening form, a questionnaire regarding cognitions about sleep, and an online sleep journal for 2 weeks. During the first session, the trajectory and goals are discussed and specified. Then, the participants’ biological rhythm is discussed, specifically taking into account the irregular work shifts. Targeted information and tips about the irregular rhythm and sleep in general are also given by the coach. During the second session, the previous exercises are discussed. Thereafter, practical tips regarding lifestyle, sleeping behavior, and relaxation are given. It is also evaluated whether a third session is needed. During the third session, exercises that need follow-up are discussed, as well as applications of the techniques in the future.

The intervention will be implemented throughout a period of 3 months, and thereafter may continue at the discretion of participating individuals and departments within the hospital.

#### Study design

A prospective study design is used to investigate the effect of the PerfectFit@Night intervention on sleep, fatigue, and recovery. The intervention is implemented at different hospital departments, making randomization at individual level not possible. Randomization at the work department was not possible, because whether and when the interested departments start PerfectFit@Night is highly dependent on the developments regarding the COVID-19 pandemic. Since randomization at individual level nor work department is possible, the intervention is implemented across at least six departments over a 3 months period, whereby departments move from the control condition to the intervention condition. In this phased pre-post approach within each department there are two periods of data collection before the start of the intervention (1 month and right before the start of the intervention) and two periods of data collection thereafter (3 months and 6 months after the start of the intervention).

The study has been approved by the Medical Ethical Review Committee of the Erasmus University Medical Center (Erasmus MC) and registered in the Netherlands Trial Register under trial number NL9224.

### Study population

The study population consists of all healthcare workers working night shifts from at least six different departments of the Erasmus University Medical Center in the Netherlands. The participants that completed the baseline questionnaires were asked to fill out a short questionnaire before the start of the intervention period, and follow-up questionnaires after three and six months after the start of the intervention (i.e., a total of four measurements).

### Sample size

A power calculation was conducted to determine the sample size. The calculation is based on the number of night workers needed to identify a medium effect-size (at least 0.27) in sleep quality, as measured with the Bergen Shift Work Sleep Questionnaire (BSWSQ) [26]. A previous study among nurses working night shifts reported a mean sleep quality of 1.75 with a standard deviation (SD) of 1.26 [26]. The design of the study is a before-after study with four measurements (two before and two after the intervention period). Assuming equal within- and between-person variance with an ICC of 0.05 for clustering at the department level, and an average cluster size of 20, five departments should be included to obtain a power of 0.80 at a significance level of 0.05. This means 100 participants should be included in total. Taking into account an initial participation of 70% and a loss-to-follow-up of 30%, the number of night workers needed at baseline is 204. It could be in practice that cluster sizes are larger than described in this power calculation. This will not have any consequences as we applied the most conservative sample size within this prospective study design with repeated measurements.

### Outcome measures

The outcomes are assessed using a questionnaire which is sent to the night workers of the participating departments both digitally and on paper.

#### Primary outcomes

The primary outcome measures are sleep, fatigue, and need for recovery, which are measured at all four time points.

##### Sleep

Sleep is measured using the Bergen Shift Work Sleep Questionnaire (BSWSQ), which is developed to assess discrete sleep problems related to different work shifts and rest days in shift workers [26]. The questionnaire consists of seven items and is based on the clinical symptoms of insomnia and fatigue/sleepiness, as described in the Diagnostic and Statistical Manual of Mental Disorders (DSM-IV). There is a distinction between insomnia items and fatigue/sleepiness items. The items can be answered on a five-point scale ranging from “never” to “always” for each shift type and for rest days separately. The BSWSQ provides a global score of sleep problems for each shift type. The score concludes insomnia as ‘unfavorable’ when at least one of the insomnia items and at least one of the fatigue/sleepiness items was scored unfavorable. The questionnaire demonstrates good reliability (interclass correlation of 0.69–0.75), and convergent and discriminant validity with other scales [26]. The BSWSQ does not provide information on bed time, sleep latency, wake-up time, amount of hours asleep, sleep medication, and the rating of subjective global sleep quality. These factors are included in the Pittsburgh Sleep Quality Index (PSQI), which assesses sleep disturbances during the past month [24]. Therefore, the BSWSQ is supplemented with the items of the PSQI concerning these factors. The entire PSQI is a widely used measure of sleep quality, with a high reliability of Cronbach’s alpha of 0.80 for the global PSQI score [27]. Also, correlations between PSQI global scores and related constructs exceeds *r* = 0.69, which shows a good construct validity [27].

##### Fatigue

To assess fatigue, the Short Fatigue Questionnaire (SFQ) [25] is used, which is a shortened version of the Checklist Individual Strength (CIS) [28]. The items are “I feel tired”, “I feel physically exhausted”, “I feel fit”, and “I get tired easily”. The questions can be answered on a seven-point scale, ranging from “yes, that is true” to “no, that is not true”. A higher score indicates more fatigue. The Cronbach’s alpha for the SFQ is high, namely 0.72–0.90 [29].

##### Need for recovery

To measure the participants’ need for recovery, the Need for Recovery Scale of the Dutch Questionnaire on the Experience and Evaluation of Work (Dutch abbreviation: VBBA 2.0) is used [30]. The Need for Recovery scale consists of six items concerning the ability to relax, and fatigue and tiredness after work, such as “I find it difficult to relax at the end of a working day”. The items can be answered on a four-point scale ranging from “never” to “always”. The Need for Recovery scale has shown good reliability (*α* = 0.86–0.88), construct validity, and sensitivity to change in The Netherlands [30–32].

#### Secondary outcomes

Secondary outcomes are assessed three times, at the first baseline measurement and at three and six months follow-up.

##### Sleep hygiene

Six self-constructed questions are asked regarding sleep hygiene, e.g. about taking naps during nights shifts, and taking into account what to eat and drink during and after night shifts. The items can be answered on a four-point scale ranging from “never” to “always”.

##### Sustainable employability

Sustainable employability is measured using three items of the Work Ability Index (WAI) [33]. The sub-items of the WAI can be used as a simple indicator for assessing the status and progress of work ability. Therefore, the first item of the WAI is included, which asks the question “Assume that your work ability at its best has a value of 10 points. How many points would you give your current work ability?” that can be answered on a scale ranging from 0 to 10. Additionally, two items on the own rating of work ability related to physical and psychological job demands are asked. These items can be answered on a five-point scale ranging from “very good” to “very bad”.

##### Dietary intake

Assessment of the dietary intake is focused on the consumption of beverages and snacks during night shifts generally over the past month. Participants are asked to indicate the amount of water, caffeinated coffee, caffeinated tea, and energy drinks they consumed, and whether they ate pre-specified snacks. The snacks are categorized, based on the revised version of the Fat List [34]. For each type of snack, it is asked whether this was eaten “never”, “sometimes”, “often” or “always”.

##### General health status

is measured subjectively using a single item of the RAND-36 Health Survey on self-perceived health status [35]. The answer options range from “very good” to “very bad”.

#### Covariates

A number of covariates are measured once at baseline.

##### Individual characteristics

Demographic characteristics such as age, gender, and household composition are collected, as well as work factors such as job title, number of years in night work, frequency of night shifts, and working hours.

##### Chronotype

To assess whether de participants are morning, evening, or intermediate type persons, the night workers are asked whether they consider themselves “definitely a morning person”, “more a morning than evening person”, “more an evening than morning person”, or “definitely an evening person”. A single question regarding self-reported chronotype has shown to be in excellent agreement with chronotype assessment based on sleep times [36].

##### Body mass index (BMI)

The participants are asked to state their height and weight. BMI will be calculated by dividing weight (in kilograms) by the square of height (in meters).

##### Physical activity

Physical activity is assessed by two questions regarding the amount of days a week an individual spends more than 30 moderate and high intensity active minutes in leisure time in general.

##### Psychological job demands

Three items of the Job Content Questionnaire (JCQ) are used to assess psychological job demands. The items measure how fast, much, and hard an individual’s work is [37]. The questions can be answered on a five-point scale ranging from “never” to “always”. The Cronbach’s alpha for the psychological job demands scale is 0.63, which indicates an acceptable reliability [37].

##### Job autonomy

Another five items of the JCQ are used to assess autonomy at work [37]. These items concern decision making, deciding on the order and speed of conducting tasks, having to find solutions, and being able to take time off. The five items are supplemented with two items on the influence on work rosters, and the opportunity to take breaks. The questions can be answered on a five-point scale ranging from “never” to “always”.

##### Social support at work

To assess social support at work, four items of the Copenhagen Psychosocial Questionnaire (COPSOQ) are used [38]. The items concern questions about help and support from colleagues and supervisors, and about how often colleagues and supervisors are willing to listen to work-related problems. The items can be answered on a five-point scale ranging from “never” to “always”. The four items have a test-retest reliability of 0.70–0.73, indicating an adequate reliability [39].

##### Emotional strain

Three items of the COPSOQ are used to assess emotional strain of the job [38]. These items concern emotionally difficult situations, emotional demands, and emotional involvement with work.

##### Rewards

Another three items of the COPSOQ are used to assess appreciation, recognition, and respect of the management, as well as fair treatment at work [38]. The items can be answered on a five-point scale ranging from “to a very small extent” to “to a very large extent”.

##### Rosters

Five self-constructed items are used to get insight in the rosters of the night workers and how satisfied they are with the rostering process. Three items concern the roster process (e.g., whether the rosters are known in a timely manner), which can be answered on a four-point scale ranging from “never” to “always”. Two items concern the questions whether complaints about the rosters are taken seriously, and whether the night worker wants to have more say in the rosters. These two items can be answered with “yes”, “no”, or “not applicable”.

##### Department characteristics

To be able to test for differences in outcomes between departments, information on the department the employee works at and the amount of night workers at that department are collected.

### Effect evaluation

Baseline characteristics of the participants and of the departments will be summarized using descriptive statistics to detect possible selection bias and lack of balance between departments. To evaluate the effectiveness of the intervention, linear mixed effects models will be applied for scale-scores, and generalized linear mixed models for dichotomous outcomes. These statistical methods take into account the repeated measures of the study design. The analyses will be adjusted for important confounders based on the individual participants and the department, and both statistical tests and confidence intervals will be calculated (with a significance level of 5%). The difference in starting point across the departments and the multilevel structure of the departments will be taken into account. In order to analyze whether the (in)effectiveness of the intervention is moderated by the implementation factors, the linear and generalized mixed models will be expanded with the process factors that emerge from the process evaluation to evaluate whether higher uptake and quality of implementation is associated with higher effect size.

### Process evaluation

The implementation process will be evaluated after the intervention period by assessing the seven components of the framework of Steckler and Linnan [40]. These are 1) recruitment: description of the procedures used to approach and attract hospital workers with night shifts to take part in the intervention; 2) reach: the proportion of hospital workers with night shifts that actually participated in one of the intervention elements; 3) dose delivered: the delivery of all intervention elements as described in the protocol; 4) dose received (compliance): the proportion of night workers that received all intervention elements; 5) fidelity: the extent to which the intervention is implemented following the intervention protocol and the implementation plan; 6) satisfaction: satisfaction of the night workers and other stakeholders about the PerfectFit@Night intervention; 7) context: the barriers and facilitators that might hamper or enhance implementation. Both quantitative and qualitative data will be collected. Quantitative data can be, for example, records of the participation in the workshop, the amount of participants that started and completed the e-learning, usage of the powernap bed and sleep coaching, and reports from the questionnaire about satisfaction with the intervention elements. Qualitative data will be collected via interviews with night workers and focus groups. Interviews will be conducted with at least two night workers from each participating department, and other stakeholders (i.e., managers, human resources) 3 months after enrolment in the intervention. The interviews will be one-on-one following a semi-structured protocol, and they will be audiotaped and transcribed. To identify common themes, two project members will analyze the first four transcripts separately. This ensures both consistent and robust coding following the process evaluation framework. Qualitative, open-ended questions from the questionnaires will be coded manually in the same manner as the interviews, and logbook data will be grouped per department to form a chronological list of events. Additionally, two focus group discussions with four team managers of different departments will be organized.

Reach, dose received, and dose delivered will be assessed based on descriptive statistics of the quantitative data. Questionnaires, interviews, and logbooks will be used to study fidelity, satisfaction, and context.

### Economic evaluation

To help employers and managers to understand the possible financial consequences or benefits of the implementation of PerfectFit@Night, cost-benefit analyses from the employers perspective will be conducted if the intervention has been proven effective. To perform the economic analysis, three measures are included in the study.

#### Intervention costs

These costs are related to the development and implementation of the intervention. They also include labor costs of the intervention staff. Some intervention elements, such as the powernap beds, have already been purchased by the departments. Therefore, these elements are not taken into account in the economic evaluation.

#### Productivity loss

Productivity costs is measured using costs due to absenteeism (sickness) and due to presenteeism (loss of productivity at work). Absenteeism is measured using three questions regarding the sickness absence of the past month. To measure productivity, two questions of the Productivity and Disease Questionnaire (PRODIQ) are asked [41]. These questions concern the amount of work done during the last work day compared to a general work day (on a scale from 0 to 10), and the reason for not being 100% productive.

## Discussion

Different studies on interventions to enhance health and sustainable employability of healthcare workers with night shifts have already been conducted, but research on the effectiveness of these measures is rare. The current study described the integrated intervention PerfectFit@Night, consisting of a set of elements focusing on individual behavior with respect to lifestyle and working conditions. Both the effectiveness of the intervention on sleep, fatigue, and recovery, and the implementation process will be evaluated. Cost-benefit analyses from the employers perspective will be conducted to understand the possible financial consequences or benefits of the implementation of PerfectFit@Night.

### Strengths and limitations

The first strength of this intervention is its integrative character. The intervention is a combination of universal prevention and indicated prevention, and it targets both individuals and the work environment. Second, the evaluation of both effectiveness and implementation makes it possible to distinguish between theory and program failure.

The study also has some limitations. First, the integrated nature of the intervention makes it impossible to make conclusions about the effects of each individual intervention element. Second, it depends on the willingness of the department heads to invest in the powernap beds and healthy nutrition. Third, whether and when the interested departments start the PerfectFit@Night is highly dependent on the developments regarding the COVID-19 pandemic, making randomization at the individual and work department not possible. Last, the time frame of the intervention leads to the inability to draw conclusions on the long-term effects.

## Conclusion

The PerfectFit@Night intervention aims to improve sleep, fatigue, and recovery of night workers in the healthcare sector by introducing an intervention program which combines universal and indicated prevention targeting both individuals at the workplace as well as the work environment. Both the effectiveness, implementation process, and cost-benefit of the PerfectFit@Night intervention will be evaluated.

## Data Availability

Not applicable. No quantitative data has been collected.

## Abbreviations

WHO: World Health Organization
ASE: attitude, social influence, and self-efficacy model
ANGELO: Analysis Grid for Environments Linked to Obesity framework
PSQI: Pittsburgh Sleep Quality Index
SFQ: Short Fatigue Questionnaire
CGTi: cognitive behavioral therapy insomnia
BSWSQ: Bergen Shift Work Sleep Questionnaire
DSM-IV: Diagnostic and Statistical Manual of Mental Disorders
CIS: Checklist Individual Strength
VBBA: Dutch abbreviation for Dutch Questionnaire on the Experience and Evaluation of Work
WAI: Work Ability Index
BMI: Body Mass Index
JCQ: Job Content Questionnaire
COPSOQ: Copenhagen Psychosocial Questionnaire
PRODIQ: Productivity and Disease Questionnaire
